# The Mitigating Effect and Mechanism of Polydeoxyribonucleotide Against Zoledronic Acid-Induced Growth Suppression of Human Gingival Fibroblasts

**DOI:** 10.3390/ijms262311367

**Published:** 2025-11-24

**Authors:** Shailashree Pachhapure, Young-Min Shin, Duk Gyu Kim, Dong-Rak Choi, Jong-IL Yun, Jae-Hong Kim, Byeong-Churl Jang

**Affiliations:** 1 Department of Molecular Medicine, School of Medicine, Keimyung University, Daegu 42601, Republic of Korea; shailashree@kmu.kr; 2 Department of Dentistry, School of Medicine, Dongsan Hospital, Keimyung University, Daegu 42601, Republic of Korea; shinym@dsmc.or.kr; 3 Zerone Cellvane Inc., Cheonan 31035, Republic of Korea; dgkim@zeronecv.com (D.G.K.); drchoi@zeronebio.com (D.-R.C.); 4 Yeon Dental Clinic, Seoul 04363, Republic of Korea; oralyun@naver.com; 5 Dental R&D Center, Zerone Cellvane Inc., Seoul 04363, Republic of Korea; flavan@naver.com; 6 Seoul Top Dental Clinic, Namyangju-si 12232, Republic of Korea

**Keywords:** HGF-1, zoledronic acid, PDRN, TBK1, PKB

## Abstract

Zoledronic acid (ZA), a nitrogen-containing bisphosphonate, is widely used to treat osteoporosis and bone metastases. However, its clinical application is limited by adverse effects, notably bisphosphonate-related osteonecrosis of the jaw (BRONJ), which is associated with cytotoxicity in oral mucosal cells. Polydeoxyribonucleotide (PDRN), a salmon sperm-derived DNA polymer with regenerative and anti-inflammatory properties, has shown therapeutic potential in tissue repair; however, its ability to mitigate ZA-induced cytotoxicity remains poorly understood. Here, we investigated the molecular mechanisms of ZA-induced toxicity in HGF-1 cells, a human gingival fibroblast line, and evaluated the protective effects of PDRN. ZA treatment (50 µM, 48 h) significantly inhibited HGF-1 cell growth, accompanied by reduced phosphorylation of protein kinase B (PKB) and signal transducer and activator of transcription 3 (STAT-3), along with increased phosphorylation of TANK-binding kinase 1 (TBK1). TBK1 silencing restored cell growth under ZA exposure, whereas silencing PKB or STAT-3 further suppressed cell growth even without ZA. Co-treatment with PDRN (100 µg/mL) effectively prevented and reversed ZA-induced HGF-1 cytotoxicity. Mechanistically, PDRN inhibited ZA-induced TBK1 phosphorylation and partially restored PKB phosphorylation, though it did not reverse the reduction in p-STAT-3. Additionally, ZA significantly elevated intracellular reactive oxygen species (ROS) levels at 8 h, which were attenuated by PDRN. The antioxidant N-acetylcysteine (NAC) similarly reduced ZA-induced ROS and p-TBK1 levels and improved cell growth, although it had limited effects on p-PKB at 8 h. Importantly, delayed PDRN treatment following ZA exposure reversed ZA-induced cell growth inhibition and TBK1 activation in a dose- and time-dependent manner. In summary, these findings demonstrate that ZA suppresses HGF-1 cell growth through ROS production, TBK1 activation, and inhibition of PKB and STAT-3, whereas PDRN counteracts these effects primarily by suppressing TBK1 activation and oxidative stress.

## 1. Introduction

Zoledronic acid (ZA) is a nitrogen-containing bisphosphonate extensively used in clinical practice to manage various bone diseases, including osteoporosis, Paget’s disease, and bone metastases [1]. It acts by inhibiting osteoclast-mediated bone resorption, thus enhancing bone mineral density and reducing the risk of fractures [2]. Despite its efficacy in strengthening bones, ZA is associated with significant adverse effects, notably osteonecrosis of the jaw (ONJ), a severe and often debilitating condition characterized by necrotic lesions in the jawbone [3]. This adverse effect highlights the need to identify new drugs or natural substances capable of suppressing ZA-induced ONJ.

ZA’s toxicity to oral mucosa cells, especially human gingival fibroblasts (HGFs), is likely to play a significant role in the development of ONJ. Mounting evidence indicates that ZA inhibits the proliferation and induces the apoptosis of HGFs [4]. Up to date, the molecular and signaling mechanisms by which ZA has growth-suppressive and/or apoptosis-inducing effects on HGFs include inhibition of mevalonate pathway, activation of caspases, inhibition of phosphoinositide 3 kinase (PI3K)/protein kinase B (PKB) signaling, down-regulation of signal transducer and activator of transcription 3 (STAT-3), production of reactive oxygen species, and mitochondrial dysfunction [5].

Polydeoxyribonucleotide (PDRN) is a salmon sperm-derived DNA polymer that has emerged as a promising regenerative agent due to its tissue-repairing, pro-angiogenic, and anti-inflammatory properties [6,7,8]. Research further demonstrates that PDRN increases cell proliferation and survival while decreasing cell apoptosis [6], suggesting its potential to protect cells from ZA-induced cytotoxicity.

Until now, however, the molecular and signaling targets mediating ZA-induced toxicity of HGFs have not been completely explored. Moreover, PDRN regulation of ZA-induced toxicity of HGFs is not fully understood. HGF-1 is a cell line exhibiting fibroblast morphology isolated from the human gingiva [7]. In this study, we investigated how ZA affects the growth of HGF-1 cells and whether PDRN mitigates this effect.

## 2. Results

### 2.1. Treatment with ZA at 50 µM Markedly Reduces the Growth of HGF-1 Cells

**Figure 1:**
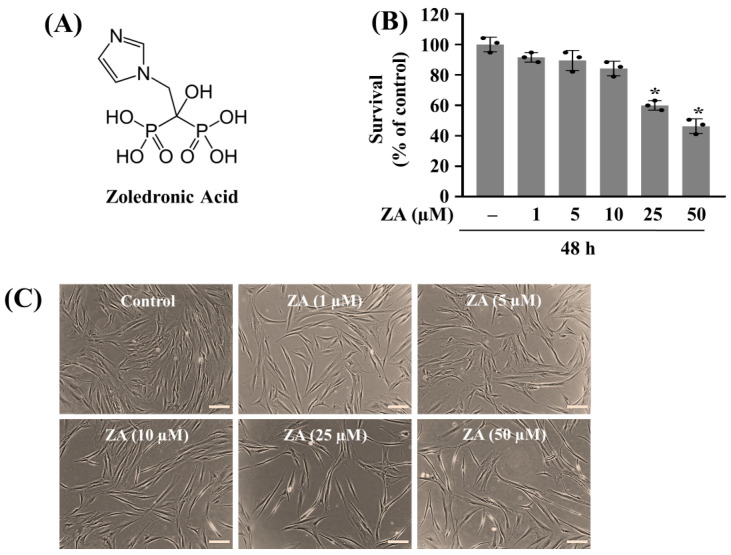
Effect of zoledronic acid (ZA) on the growth (survival) of HGF-1 cells. (**A**) The chemical structure of ZA. (**B**) HGF-1 cells were treated with ZA at the designated concentrations for 48 h. The number of surviving cells was measured using cell count assay. The cell count assay was performed in triplicate. Data are means ± standard error (SE) of three independent experiments. * *p* < 0.05 compared to the value of ZA or control for the indicated time. (**C**) A representative image of morphological changes in the conditioned cells in (**B**) Scale bar = 100 µm.

Initially, we investigated the treatment effect of ZA (Figure 1A–C) at 1, 5, 10, 25, and 50 µM on the growth of HGF-1 cells for 48 h using cell count analysis and a phase-contrast microscopic observation. As shown in Figure 1B, ZA treatment led to a concentration-dependent decrease in the growth of HGF-1 cells. Compared to control, ZA treatment at 1 or 5 µM decreased cell survival only slightly, and a more pronounced reduction of HGF-1 cells was observed at 10 µM. Treatment with ZA at 25 µM and 50 µM led to a significant decrease in cell growth. Figure 1C are the micrographs showing representative phase-contrast images of HGF-1 cells under control conditions (no ZA) and following 48 h treatment with 1, 5, 10, 25, and 50 µM ZA. Control cells exhibited a typical spindle-shaped fibroblast morphology, forming a confluent layer. Treatment with ZA at 1 µM or 5 µM caused minimal changes in HGF-1 cells; most cells retained their elongated shape and remained well attached, indicating relatively low toxicity at these concentrations. ZA treatment at 10 µM apparently led to a moderate decrease in cell density; some cells showed early signs of morphological changes such as rounding or slight shrinkage. Treatment with ZA at 25 µM or 50 µM resulted in a marked reduction in the cell number, and the remaining cells appeared more rounded and shrunken, consistent with a loss of viability. These data suggest that ZA has an apparent dose-dependent inhibitory effect on the growth of HGF-1 cells, with substantial cytotoxicity and pronounced morphological signs of cellular damage evident at 25 µM and above. Due to the strongest cytotoxic effect, we selected the 50 µM concentration of ZA for further studies.

### 2.2. Treatment with ZA at 50 µM Significantly Decreases Phosphorylation of PKB and STAT-3 While Increasing That of TBK1 in HGF-1 Cells

**Figure 2:**
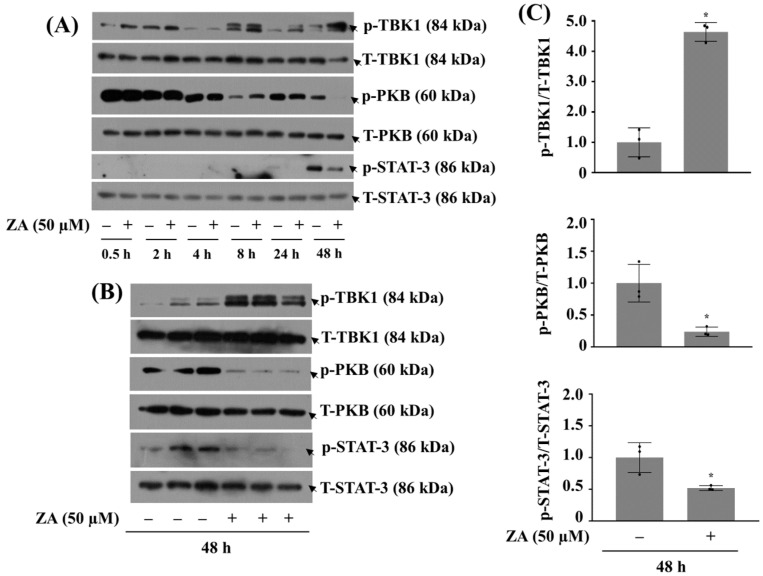
Effect of ZA on the phosphorylation and expression levels of TBK1, PKB, and STAT-3 in HGF-1 cells. (**A**) HGF-1 cells were treated without or with ZA (50 µM) for the indicated times. At each time point, whole-cell lysates were prepared and analyzed by Western blotting to measure the phosphorylation and total expression levels of TBK1, PKB, and STAT-3. (**B**) HGF-1 cells were treated without or with ZA (50 µM) in triplicate for 48 h. Whole-cell lysates were prepared and analyzed by Western blotting to measure the phosphorylation and total expression levels of TBK1 and PKB. (**C**) Densitometry analysis of (**B**). Data are mean ± SE of three independent experiments. * *p* < 0.05 compared to control.

Next, to understand potential molecular and signaling factors associated with ZA-induced growth inhibition in HGF-1 cells, we analyzed the treatment effect of ZA at 50 µM on the phosphorylation and expression levels of PKB, STAT-3, and TBK1 in HGF-1 cells over time (0.5~48 h). As shown in Figure 2A, compared to control, levels of p-TBK1 in HGF-1 cells treated with ZA remained relatively low or only slightly increased at early time points (0.5, 2, 4, 8, 24 h). Apparently, treatment with ZA at 48 h led to a marked increase in levels of p-TBK1 compared to earlier time points, indicating a strong activation of TBK1 in response to prolonged ZA exposure. In HGF-1 cells, levels of p-PKB were detectable at early time points but gradually decreased as ZA treatment continued. Treatment with ZA at 24 and 48 h caused a notably reduced levels of p-PKB, reflecting a significant suppression of PKB activity under prolonged ZA exposure. Levels of p-STAT-3 in HGF-1 cells exposed to ZA were less pronounced at times tested herein, compared to the strong changes seen in levels of p-TBK1 or p-PKB. The absence or low levels of p-STAT-3 at early time points and of p-TBK1 at 4 h may be attributed either to the transient nature of these phosphorylation events or to their inherently low basal activity in the absence of treatment. Total expression levels of TBK1, PKB, and STAT-3 remained relatively constant across all time points, suggesting that the observed increase in p-TBK1 is not due to the de novo protein expression (synthesis) but rather the enhanced phosphorylation from preexisted protein. As further illustrated in Figure 2B, triplicate experiments revealed that 48 h ZA treatment significantly reduced the phosphorylation of PKB and STAT-3, while enhancing the phosphorylation of TBK1 in HGF-1 cells. Corresponding densitometry analyses are presented in Figure 2C, showing the relative phosphorylation and expression levels of TBK1, PKB, and STAT-3, each normalized to their respective total protein levels.

### 2.3. Silencing of TBK1 Partially Blocks ZA-Induced HGF-1 Cytotoxicity

**Figure 3:**
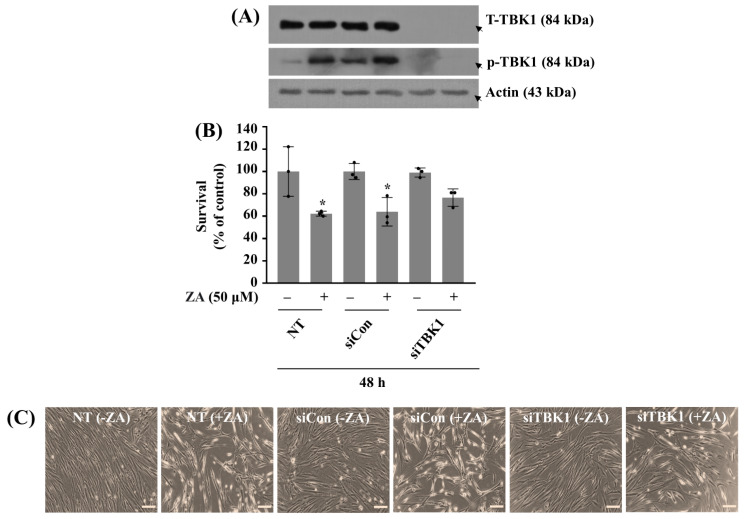
Effect of TBK1 knockdown on the growth of HGF-1 cells and the expression and phosphorylation levels of TBK1 under ZA exposure. (**A**,**B**) HGF-1 cells were transfected with 200 pM of control siRNA (siCon) or TBK1 siRNA (siTBK1) for 48 h. The transfected cells were then treated without or with ZA (50 µM) for an additional 48 h. Whole-cell lysates were then prepared and analyzed by Western blotting to measure the total expression and phosphorylation levels of TBK1 (**A**). The number of surviving cells was measured using cell count assay. The cell count assay was performed in triplicate. Data are means ± SE of three independent experiments. * *p* < 0.05 compared to the value of ZA or control for the indicated time (**B**). (**C**) A representative image of morphological changes in the conditioned cells in (**B**). Scale bar = 100 µm.

Next, we explored the role of increased TBK1 phosphorylation (activity) in ZA-induced growth suppression of HGF-1 cells using siRNA transfection, Western blotting, and cell count assay. As shown in Figure 3A, both total TBK1 expression and p-TBK1 levels in HGF-1 cells transfected with siTBK1 for 48 h were markedly reduced compared to non-transfected (NT) or control siRNA (siCon) cells regardless of ZA absence or presence, confirming the siTBK1 transfection efficiency. Notably, in the presence of 50 µM ZA, NT and siCon cells exhibited a significant decrease in cell survival (Figure 3B,C). However, siTBK1 cells treated with ZA displayed a higher survival rate than siCon or NT cells exposed to ZA. These results demonstrate that knockdown of TBK1 partially rescues HGF-1 cells from ZA-induced cytotoxicity and suggest that the increased TBK1 activity plays a pivotal role in mediating ZA’s HGF-1 cytotoxic effect.

### 2.4. Knockdown of PKB or STAT-3 Further Reduces the Growth of HGF-1 Cells in the Absence of ZA

**Figure 4:**
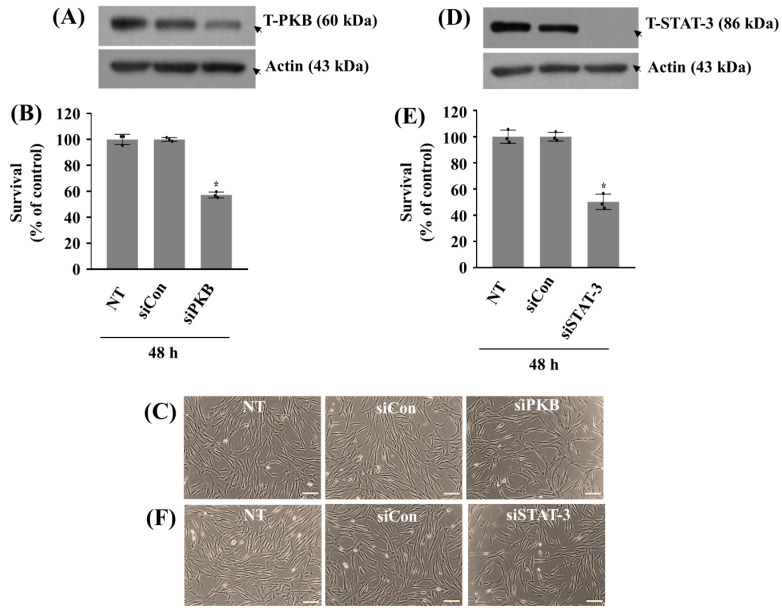
Effect of PKB or STAT-3 knockdown on the growth of HGF-1 cells and the expression levels of PKB or STAT-3 without ZA (**A**–**F**). (**A**,**B**) HGF-1 cells were transfected with 200 pM of control siRNA (siCon) or PKB siRNA (siPKB) for 48 h. Whole-cell lysates were then prepared and analyzed by Western Blotting to measure the total expression levels of PKB and Actin (**A**). The number of surviving cells was measured using cell count assay. The cell count assay was performed in triplicate. Data are means ± SE of three independent experiments. * *p* < 0.05 compared to the value of control for the indicated time (**B**). (**C**) A representative image of morphological changes in the conditioned cells in (**B**) Scale bar = 100 µm. (**D**,**E**) HGF-1 cells were transfected with 200 pM of control siRNA (siCon) or STAT-3 siRNA (siSTAT-3) for 48 h. Whole-cell lysates were then prepared and analyzed by Western Blotting to measure the total expression levels of STAT-3 and Actin (**D**). The number of surviving cells was measured using cell count assay. The cell count assay was performed in triplicate. Data are means ± SE of three independent experiments. * *p* < 0.05 compared to the value of control for the indicated time (**E**). (**F**) A representative image of morphological changes in the conditioned cells in (**E**). Scale bar = 100 µm.

Next, we probed the role of decreased PKB or STAT-3 phosphorylation (activity) in ZA-induced growth suppression of HGF-1 cells using respective siRNA transfection, Western blotting, and cell count assay. As shown in Figure 4A or Figure 4D, total PKB or STAT-3 expression levels in HGF-1 cells transfected with siPKB or siSTAT-3 for 48 h were substantially reduced compared to non-transfected (NT) or control siRNA (siCon) cells, assuring each siPKB or siSTAT-3 transfection efficiency. Of interest, siPKB or siSTAT-3 cells displayed much lower survival rate than siCon or NT cells in the absence of ZA (Figure 4B,C,E,F). These results suggest that PKB and STAT-3 act as survival factors in HGF-1 cells and their loss (inhibition) contributes to ZA’s cytotoxic effect herein.

### 2.5. Co-Treatment with PDRN at 100 µg/mL Effectively Blocks ZA-Induced Growth Inhibition and Leads to Alterations of the Increased TBK1 or Decreased PKB Phosphorylation in HGF-1 Cells

**Figure 5:**
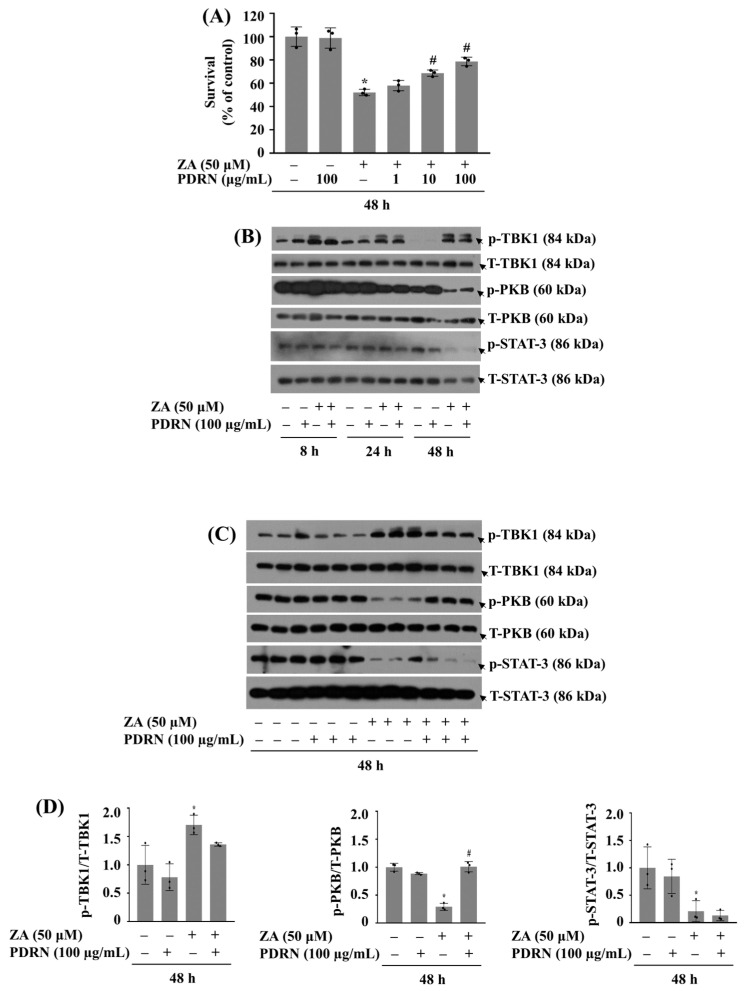
Effect of ZA and/or PDRN on the growth of HGF-1 cells and the phosphorylation and expression levels of TBK1, PKB and STAT-3. (**A**) HGF-1 cells were treated with or without ZA (50 µM) and PDRN (1, 10 or 100 µg/mL) for 48 h. The number of surviving cells was measured by cell count assay. The cell count assay was performed in triplicate. Data are means ± SE of three independent experiments. * *p* < 0.05 compared to the value of ZA or control for the indicated time. # *p* < 0.05 compared with the values of ZA treatment (with ZA and PDRN). (**B**) HGF-1 cells were treated without or with ZA in the absence or present of PDRN at the designated doses and times. At each time point, whole-cell lysates were prepared and analyzed by Western Blotting to measure the phosphorylation and total expression levels of TBK1, PKB and STAT-3. (**C**) HGF-1 cells were treated without or with ZA in the absence or present of PDRN at 100 µg/mL dose for 48 h. After 48 h, Whole-cell lysates were prepared and analyzed by Western Blotting to measure the phosphorylation and total expression levels of TBK1, PKB and STAT-3. (**D**) Densitometry analysis of (**C**). Data are mean ± SE of three independent experiments. * *p* < 0.05 compared to control.

Next, we evaluated the effect of PDRN at different concentrations (1, 10, and 100 µg/mL) on ZA-induced growth suppression of HGF-1 cells. As anticipated, compared to control (no ZA), single treatment with 50 µM ZA significantly reduced the growth of HGF-1 cells (Figure 5A). However, treatment with PDRN at 1, 10 or 100 µg/mL had a dose-dependent protective effect against ZA-induced growth suppression of HGF-1 cells. Apparently, PDRN at 100 µg/mL most strongly protected cells from ZA-induced cytotoxic effect. Notably, treatment with PDRN alone at concentrations of 1, 10, and 100 µg/mL for 48 h did not induce any detectable cytotoxic effects in HGF-1 cells, as assessed by morphological observation and cell counting. These findings confirm that the concentrations used in this study are non-toxic under the experimental conditions applied. Next, we investigated the effect of 100 µg/mL PDRN on ZA-induced alterations of TBK1, PKB, and STAT-3 phosphorylation in HGF-1 cells over time. Of note, as shown in Figure 5B, PDRN treatment partially blocked ZA-induced TBK1 phosphorylation in HGF-1 cells at times tested. In addition, PDRN treatment partially restored p-PKB levels in HGF-1 cells at 48 h. As further shown in Figure 5C,D, triplicate experiments exhibited the PDRN capability to decrease ZA-induced TBK1 phosphorylation and to inhibit the drug-induced reduction of PKB phosphorylation with no alteration of the reduced STAT-3 phosphorylation in HGF-1 cells. Total expression levels of TBK1, PKB, and STAT-3 remained constant under these experimental conditions. To investigate whether ZA-induced growth inhibition of HGF-1 cells is associated with apoptosis, we performed Annexin V-FITC/7-AAD dual staining followed by flow cytometric analysis. As shown in Supplementary Figure S1, treatment with ZA (50 µM, 48 h) induced a modest increase (~5%) in the apoptotic cell population (Q2 + Q4). Co-treatment with PDRN (100 µg/mL) slightly reduced this ZA-induced apoptosis, whereas PDRN alone did not affect basal apoptosis. These findings suggest that PDRN provides a mild protective effect against ZA-induced apoptotic cell death in HGF-1 cells.

### 2.6. PDRN’s Protective Action Against ZA-Induced Growth Suppression of HGF-1 Cells Is at Least Partly Mediated by A2AR

**Figure 6:**
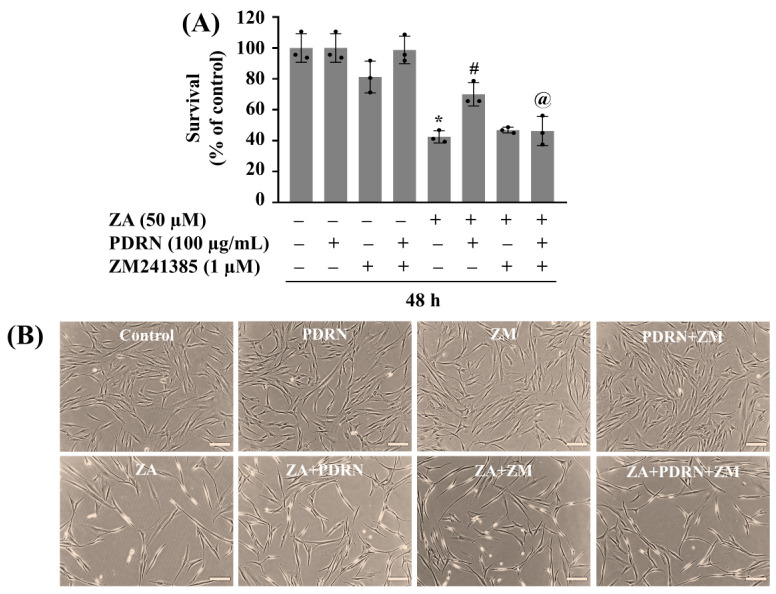
Effect of PDRN and/or ZM241385 (ZM) on ZA-induced growth suppression of HGF-1 cells. (**A**) HGF-1 cells were treated without or with ZA in the absence or presence of PDRN or ZM, an A_2_AR antagonist, at the designated concentrations for 48 h. The number of surviving cells was analyzed using cell count analysis. Data represent the mean ± SE of three independent experiments. * *p* < 0.05 compared with the values of control. # *p* < 0.05 compared with the values of ZA treatment. @ *p* < 0.05 compared with the values of ZA and PDRN treatment. (**B**) A representative image of morphological changes in the conditioned cells in (**A**). Scale bar = 100 µm.

Next, we investigated whether PDRN’s protective effect on ZA-induced cytotoxicity is mediated through the A_2_AR pathway using ZM241385, a selective A_2_AR antagonist. As shown in Figure 6A, ZA treatment significantly decreased the growth of HGF-1 cells, and treatment with PDRN at 100 µg/mL counteracted ZA’s inhibitory effect on HGF-1 cell growth. However, while ZM241385 treatment at 1 µM did not influence ZA’s HGF-1 cytotoxicity, it significantly attenuated the protective effect of PDRN on ZA-induced HGF-1 cell growth suppression. Microscopic observation further showed that ZM241385 negated PDRN’s protective effect on ZA-induced growth inhibition of HGF-1 cells (Figure 6B).

### 2.7. PDRN Effectively Reduces ZA-Induced ROS Production and Mitigates Growth Suppression of HGF-1 Cells

**Figure 7:**
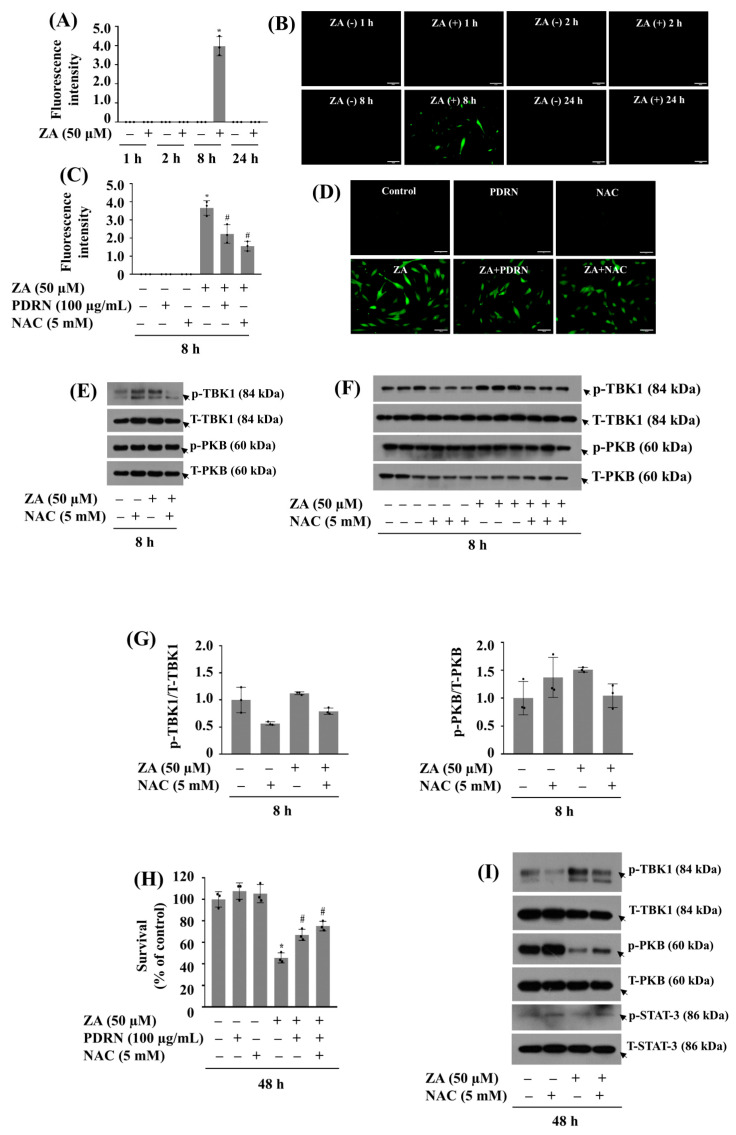

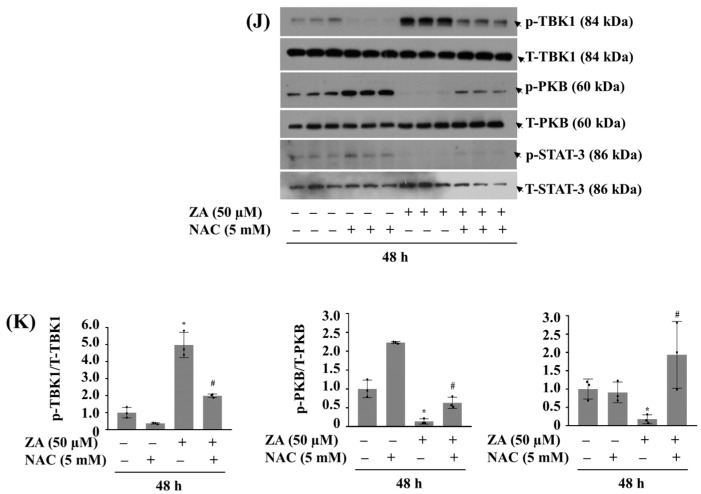
Effect of ZA and/or PDRN or NAC on ROS generation in HGF-1 cells. (**A**) HGF-1 cells were treated with or without ZA for 1, 2, 8, or 24 h. At each time point, cells were loaded with DCFH-DA and the conditioned cells’ DCF fluorescence intensity (ROS generation) was measured using fluorescence microscopy. Quantification was conducted using % intensity for each picture using image J. The DCF fluorescence is presented as mean ± standard deviation (SD) (*N* = 3) from the histogram statistics. # *p* < 0.05 vs. control (0 min). (**B**) Intracellular ROS levels of the conditioned cells in (**A**) were measured by fluorescence microscope. Scale bar = 100 µm. (**C**) HGF-1 cells were treated without or with ZA and/or PDRN or NAC at 8 h. The conditioned cells’ DCF fluorescence intensity (ROS generation) and quantification were measured using fluorescence microscopy and image J, respectively. The DCF fluorescence is presented as mean ± SD (*N* = 3) from the histogram statistics. * *p* < 0.05 compared with the values of control. (**D**) Intracellular ROS levels of the conditioned cells in (**C**) were measured by fluorescence microscope. Scale bar = 100 µm. (**E**) HGF-1 cells were treated without or with ZA (50 µM) in the absence or presence of NAC (5 mM) for 8 h. Whole-cell lysates were prepared and analyzed by Western Blotting. (**F**) Triplicate experiment of (**E**). (**G**) Densitometry analysis of (**F**). (**H**) HGF-1 cells were treated without or with ZA (50 µM) in the absence or presence of PDRN (100 µg/mL) or NAC (5 mM) for 48 h. The number of surviving cells was measured by cell count assay. The cell count assay was performed in triplicate. Data are means ± SE of three independent experiments. * *p* < 0.05 compared to the value of ZA or control for the indicated time. # *p* < 0.05 compared with the values of ZA treatment. (**I**) HGF-1 cells were treated without or with ZA (50 µM) in the absence or present of NAC (5 mM) for 48 h. Whole-cell lysates were prepared and analyzed by Western Blotting. (**J**) Triplicate experiment of (**I**). (**K**) Densitometry analysis of (**J**).

Next, we investigated whether ZA induces oxidative stress by measuring intracellular ROS levels in HGF-1 cells over time. As shown in Figure 7A, ZA treatment for 1, 2 or 24 h had little effect on ROS production in HGF-1 cells, treatment with ZA for 8 h led to a significant increase of ROS levels in these cells. Confocal microscopic observations further demonstrated that ZA treatment at 8 h markedly induced green fluorescence (ROS production) in HGF-1 cells (Figure 7B). We then examined whether PDRN inhibits ZA-induced ROS production in HGF-1 cells. For this, N-acetyl L-cysteine (NAC)_,_ a known ROS scavenger [8], was included as a positive control. As shown in Figure 7C, co-treatment with PDRN at 100 µg/mL or NAC at 5 mM significantly attenuated ZA-induced ROS production in HGF-1 cells. Fluorescence microscopic observations also exhibited the capability of PDRN or NAC to inhibit ROS generation induced by ZA in HGF-1 cells (Figure 7D). Next, to see any link between ROS production and TBK1 phosphorylation in response to ZA exposure, we examined the effect of NAC on TBK1 phosphorylation in HGF-1 cells exposed to ZA for 8 h. As shown in Figure 7E, co-treatment with NAC appeared to partially reduce ZA-induced TBK1 phosphorylation, although both p-TBK1 and T-TBK1 bands were decreased in this representative blot. To clarify this, we performed triplicate experiments with densitometry quantification (Figure 7F,G), which confirmed that NAC specifically and partially reduced ZA-induced TBK1 phosphorylation when normalized to T-TBK1 levels. Moreover, co-treatment with NAC significantly attenuated ZA-induced HGF-1 cell growth inhibition and TBK1 phosphorylation at 48 h (Figure 7H,I). Notably, NAC treatment also significantly blocked ZA-induced reduction of PKB and STAT-3 phosphorylation at 48 h in HGF-1 cells. As further demonstrated in Figure 7J,K, triplicate experiments confirmed that NAC effectively attenuated ZA-induced phosphorylation of TBK1 and prevented the drug-induced reduction in phosphorylation levels of PKB and STAT-3 in HGF-1 cells. Notably, the total protein levels of TBK1, PKB, and STAT-3 remained unchanged under these experimental conditions.

### 2.8. 48 H Post-Treatment of PDRN Mitigates the Growth Inhibition of HGF-1 Cells and TBK1 Phosphorylation Induced by 48 H (Pre)Treatment with ZA

**Figure 8:**
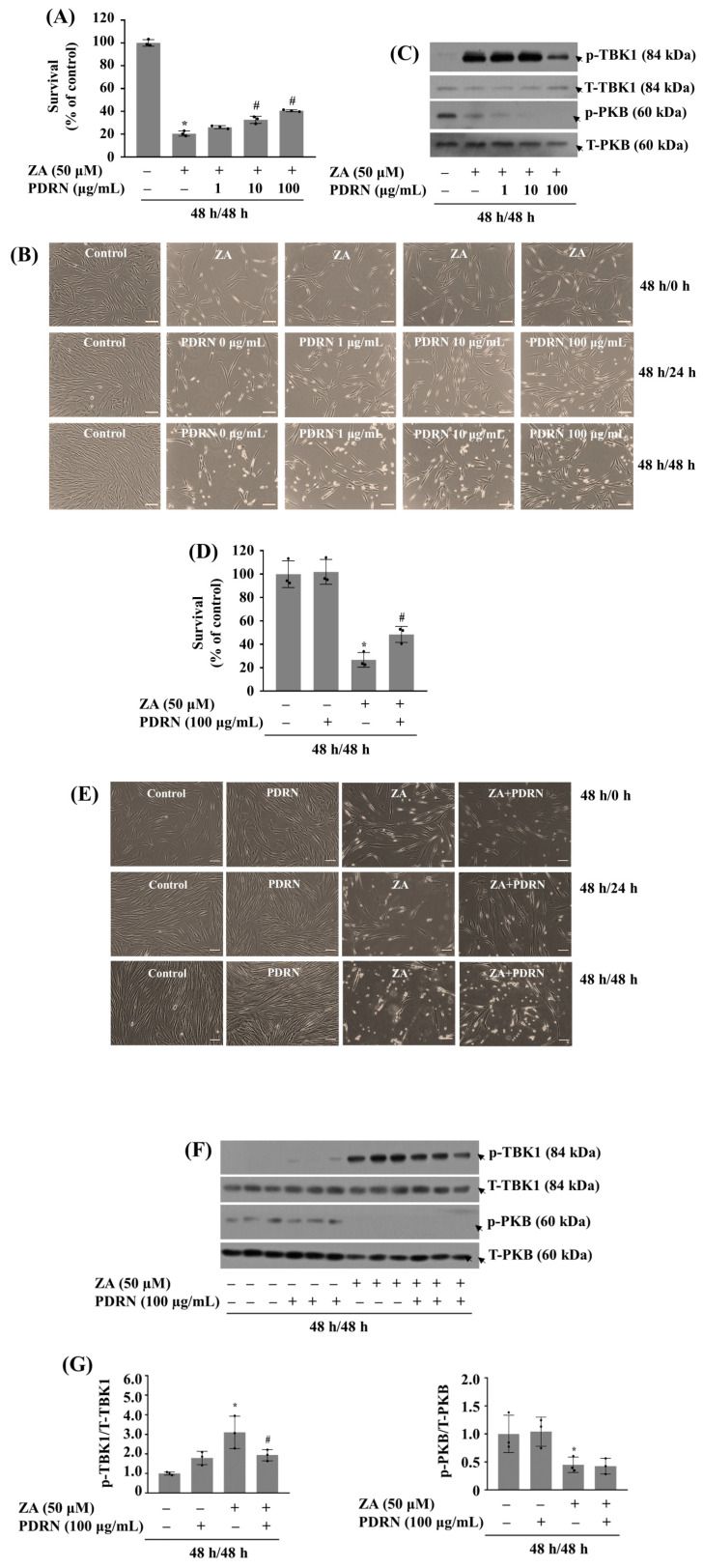
Therapeutic potential of PDRN against the growth inhibition of HGF-1 cells and TBK1 phosphorylation induced by the prior ZA treatment for 48 h. (**A**) HGF-1 cells were initially treated without or with ZA for 48 h. Cells were then incubated without or with PDRN at the indicated concentrations in the absence of ZA for an additional 48 h. The number of surviving cells was analyzed using cell count assay. The cell count assay was performed in triplicate. Data are means ± SE of three independent experiments. * *p* < 0.05 compared to the control at the indicated concentrations. # *p* < 0.05 compared with the values of ZA treatment. (**B**) A representative image of morphological changes in the conditioned cells in (**A**). Scale bar = 100 µm. (**C**) HGF-1 cells were initially treated without or with ZA for 48 h. Cells were then incubated without or with PDRN at the indicated concentrations in the absence of ZA for an additional 24 or 48 h. Whole-cell lysates were prepared and analyzed by Western Blotting to measure the phosphorylation and total expression levels of TBK1 and PKB. (**D**) Triplicate experiment of (**A**). (**E**) A representative image of morphological changes in the conditioned cells in (**D**). Scale bar = 100 µm. (**F**) Triplicate experiment of (**C**). (**G**) Densitometry analysis of (**F**).

Next, we studied the therapeutic effect of PDRN on the growth suppression of HGF-1 cells induced by 48 h pretreatment with ZA. For this, in the absence of PDRN, HGF-1 cells were first treated without or with 50 µM ZA for 48 h (to induce the cell growth inhibition by ZA). After the initial ZA treatment, cells were washed and placed in fresh medium without ZA but with PDRN at 1, 10, or 100 µg/mL for an additional 48 h (to test whether PDRN can modulate the cytotoxic damage triggered by the prior ZA treatment). Remarkably, as shown in Figure 8A, 48 h post-treatment of PDRN at 100 µg/mL exhibited the most substantial rescue or recovery against the cytotoxic damage elicited by the prior ZA treatment. Microscopic observation further underscored PDRN’s therapeutic potential for mitigating the toxic effect of HGF-1 cells induced by the prior ZA treatment (Figure 8B). Furthermore, 48 h post-treatment of 100 µg/mL PDRN strongly blocked the hyper phosphorylation of TBK1 in HGF-1 cells induced by the prior ZA treatment (Figure 8C). In contrast, 48 h post-treatment of 100 µg/mL PDRN did not affect the down-regulation of PKB phosphorylation in HGF-1 cells induced by the prior ZA treatment; rather it reduced levels of p-PKB. Total expression levels of TBK1 and PKB remained largely unchanged under these experimental conditions. As further shown in Figure 8D–G, triplicate experiments demonstrated PDRN’s therapeutic potential for mitigating the toxic effect of HGF-1 cells and TBK1 hyper phosphorylation induced by the prior ZA treatment with no change of the reduced PKB phosphorylation.

## 3. Discussion

ZA is a powerful bisphosphonate used in treating bone diseases, but its benefits come with a significant drawback: cytotoxicity in non-target cells such as human gingival fibroblasts (HGFs). Damage to these cells is implicated in oral complications, including impaired wound healing and even ONJ [9]. This study demonstrates that ZA exerts a potent, dose-dependent cytotoxic effect on HGF-1 cells, and the drug’s cytotoxic effect is due to ROS production, TBK1 activation, and inhibition of PKB and STAT-3. Our data further reveals the capability of PDRN to both prevent and rescue or reverse ZA-induced HGF-1 cytotoxicity by controlling TBK1, PKB, and oxidative stress. To the best of our knowledge, this is the first study to demonstrate that ZA induces TBK1 activation in human gingival fibroblasts. Although ZA has previously been shown to induce oxidative and mitochondrial stress, the mediating role of TBK1 in this process has not yet been reported. Here, we further show for the first time that PDRN inhibits ZA-induced TBK1 activation, thereby revealing a novel cytoprotective mechanism of PDRN that is distinct from its previously known A_2_A receptor–mediated effects [10].

ZA is widely recognized for its bone-protective properties, yet its adverse effects on oral mucosal cells are significant. Previous studies have reported that bisphosphonates including ZA induce cytotoxic effects on various cell types, including HGF-1 cells [11,12,13]. In agreement with this, we herein demonstrate the concentration-dependent decline of HGF-1 cell growth by ZA, particularly at 25 and 50 µM, further underscoring the drug’s detrimental effect on oral mucosal cells. Morphologically, HGF-1 cells exposed to ZA at 50 µM herein exhibit features consistent with cellular stress and apoptosis, such as rounding and shrinkage. These morphological changes are consistent with a loss of HGF-1 cell viability and structural integrity in response to ZA exposure.

ZA-induced cellular stress in the cells is likely multifactorial, involving a complex interplay of disrupted cellular signaling pathways (components) and stress responses [14]. A notable finding of the present study is ZA regulation of PKB phosphorylation in HGF-1 cells. PKB is a serine/threonine kinase activated primarily through the PI3K signaling pathway. Under normal conditions, PKB promotes cell survival, growth, and metabolism. Upon PKB activation (phosphorylation at S473), it phosphorylates various downstream targets involved in cell growth and survival [15,16,17]. Accordingly, active PKB promotes cellular proliferation, enhances gene expression that drives cell cycle progression, and inhibits apoptosis [18,19,20]. It has also been reported that PKB regulates pathways controlling glucose uptake and metabolism, which are essential for energy production and cellular repair [21]. In oral mucosal cells, especially gingival fibroblasts, sustained PKB activity is key to tissue repair and regeneration. This ensures that oral mucosal cells can respond effectively to injury and maintain the structural and functional integrity of the oral mucosa [22]. Of note, in the present study, treatment with ZA at 50 µM significantly reduces PKB phosphorylation in HGF-1 cells. Assuming that PKB phosphorylation is critical for its activation, it is speculative that ZA at 50 µM can induce the loss of active PKB and suppress the protein’s pro-survival signaling in HGF-1 cells. Further considering the present findings with experiments using siRNA that knockdown of PKB leads to a significant decrease in the growth of HGF-1 cells, it is conceivable that PKB acts as a pro-survival kinase in these cells and that the loss of active PKB contributes to ZA-induced HGF-1 cytotoxicity.

Another interesting finding of the present study is ZA regulation of TBK1 phosphorylation (activation) in HGF-1 cells. TBK1 is a serine/threonine kinase that is critical in the cellular stress response and innate immunity [23]. TBK1 is constitutively expressed in many cell types, including HGFs, where it is part of the cellular machinery that responds to stress signals [24]. Under normal conditions, TBK1 remains at a basal phosphorylation level, meaning that its catalytic activity is modest and tightly regulated. TBK1 activation is primarily controlled by the protein phosphorylation (at S172), typically at the activation loop [25]. TBK1 phosphorylation can be driven by various upstream signals that reflect cellular stress, including oxidative stress, mitochondrial dysfunction, or damage-associated molecular patterns [26,27]. In this study, ZA treatment at 50 µM leads to a robust increase in TBK1 phosphorylation in HGF-1 cells without altering its total protein expression, which indicates that ZA does not alter de novo TBK1 synthesis but instead triggers the post-translational modification of TBK1 already existed in these cells. This TBK1 hyperactivation likely serves as a signal amplifier for stress pathways in HGF-1 cells exposed to ZA, herein, ultimately leading to the drug’s cytotoxicity. This notion is supported by the present findings that silencing of TBK1 partially restores HGF-1 cell growth under ZA exposure. These results indicate that TBK1 activation is crucial for the drug’s cytotoxic effect herein.

Previous studies have reported ZA’s pro-oxidant effects on various cell types including HGFs [28,29]. It also has been shown that ZA increases ROS production in osteoclasts, leading to cell apoptosis [30]. In this study, treatment with ZA (50 µM, 8 h) leads to production of excessive ROS in HGF-1 cells. The observed inhibitory effect of PDRN on ZA-induced ROS production in HGF-1 cells herein aligns with the antioxidant properties of PDRN reported in other studies [31]. Furthermore, given that treatment with PDRN or NAC significantly inhibits ZA-induced ROS production and TBK1 phosphorylation and HGF-1 cell growth suppression, it is thus likely to be that ROS generation plays a crucial role in ZA-induced HGF-1 toxicity and that oxidative stress may lie upstream of TBK1 activation in HGF-1 cells exposed to ZA, which is, to our best knowledge, the first to report. Collectively, these results suggest that targeting ROS could be a critical strategy for mitigating ZA-induced toxicity in HGF-1 cells. This contrasts with NAC, which functions primarily as a direct scavenger of ROS and as a precursor of glutathione [32]. In comparison, PDRN appears to modulate oxidative stress indirectly through A_2_A receptor-coupled adenylyl cyclase signaling. Activation of this pathway elevates intracellular cAMP levels, leading to the activation of PKA and downstream transcription factors such as Nrf2, which in turn upregulate antioxidant enzymes including heme oxygenase-1 and superoxide dismutase [33]. Through this receptor-mediated redox signaling cascade, PDRN may contribute to the restoration of redox homeostasis, attenuate excessive ROS generation, and potentially suppress TBK1 activation. Therefore, it is plausible that PDRN does not directly scavenge ROS but rather coordinates redox balance through secondary messenger-dependent modulation of antioxidant defenses and kinase activity [32].

The loss of viable and functional HGFs is likely to be a critical factor in developing bisphosphonate-related ONJ (BRONJ), also named medication-related ONJ (MRONJ), highlighting the need for protective interventions [14]. There are a few effective treatments to mitigate ZA-induced cytotoxic effects on gingival tissues [33,34]. As mentioned above, PDRN is a biologically active DNA polymer derived from salmon sperm. Of interest, in the current study, co-treatment with PDRN at 100 µg/mL significantly attenuates ZA-induced HGF-1 cell growth suppression. Considering the present findings that PDRN further blocks ZA-induced TBK1 hyperphosphorylation in HGF-1 cells and that TBK1 knockdown partially rescues cells from ZA-induced toxicity in HGF-1 cells, it is obvious that PDRN exerts its protective effect on ZA-induced HGF-1 cytotoxicity by inhibiting TBK1 activation, which is, to our best knowledge, also the first to report. Thus, PDRN could be a promising candidate for mitigating ZA’s HGF-1 toxicity and be administered as an adjunct therapy alongside ZA or through systemic administration or local (topical) applications in the oral cavity against periodontal diseases in which ZA-induced loss of HGF-1 cells is problematic.

Mounting evidence demonstrates PDRN’s ability to promote cell proliferation and tissue repair (wound healing), stimulate angiogenesis, and attenuate inflammatory response via A_2_AR signaling [31,35]. In the present study, co-treatment with ZM241385, a selective A_2_AR antagonist, effectively abolishes the PDRN’s ability to block ZA-induced HGF-1 cell growth suppression, underscoring the involvement of A_2_AR signaling in this context.

Clinically, PDRN has been employed in the treatment of chronic wounds, pressure ulcers, and various skin lesions due to its ability to promote tissue repair, angiogenesis, and anti-inflammatory responses via activation of A_2_AR pathways. Emerging clinical and preclinical evidence also supports its application in oral medicine, where it facilitates mucosal healing and helps prevent local tissue damage, including that induced by bisphosphonates.

The results presented here not only shed light on the molecular underpinnings of ZA-induced toxicity in HGF-1 cells but also highlight the therapeutic potential of PDRN, partly to MRONJ. The present findings with the capacity of PDRN to prevent and reverse ZA-induced growth suppression of HGF-1 cells, even when applied as a post-treatment, suggest that it may offer a viable strategy for mitigating MRONJ at the oral mucosal cellular levels. Given that the clinical management of MRONJ remains challenging, targeting the cellular and molecular events, specifically, the damaged HGFs and dysregulation of TBK1 and PKB signaling, could also pave the way for novel therapeutic interventions. However, while the in vitro findings herein are promising, further in vivo studies are warranted to validate the efficacy and safety of PDRN in clinical settings. Future research should also aim to elucidate the broader network of signaling events involved in ZA-induced toxicity in not only HGF-1 cells but also other oral mucosal cells and to explore whether combinatory approaches targeting multiple pathways could enhance therapeutic outcomes.

**Figure 9:**
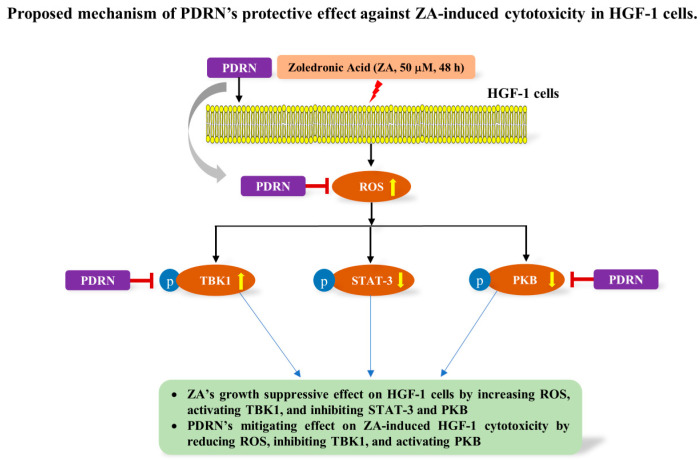
Proposed mechanism of PDRN’s protective effect against ZA-induced cytotoxicity in HGF-1 cells. Zoledronic acid (ZA, 50 μM, 48 h) increases the generation of ROS, activates TBK1, and suppresses STAT-3 and PKB, leading to cytotoxicity in HGF-1 cells. PDRN reduces ZA-induced ROS production, inhibits the activation of TBK1, and restores PKB activation, thus mitigating ZA-induced cytotoxicity.

For a clearer understanding of the general protective mechanism of PDRN, our proposed model (Figure 9) illustrates how PDRN counteracts the cytotoxic effects of ZA in HGF-1 cells. Specifically, ZA exposure markedly increases intracellular ROS levels, which in turn induce TBK1 phosphorylation and activation while concurrently suppressing the activities of PKB and STAT-3. These molecular alterations collectively contribute to the inhibition of HGF-1 cell growth and survival. PDRN exerts its protective effects primarily by attenuating ZA-induced oxidative stress, as evidenced by the reduction in intracellular ROS. At the same time, PDRN suppresses TBK1 phosphorylation and partially restores PKB activity. These combined actions likely underlie the improved cell viability and maintenance of normal cellular morphology under ZA-induced stress. Notably, the modulatory effect of PDRN on STAT-3 appears limited, suggesting that its principal protective mechanism involves regulation of the TBK1 and PKB signaling pathways. Figure 9 integrates these findings into a unified mechanistic model, highlighting the multi-targeted actions of PDRN in mitigating ZA-induced cellular damage. This conceptual framework may serve as a foundation for understanding the signaling dynamics involved and guiding future therapeutic strategies.

These findings have potential translational relevance for the prevention and treatment of MRONJ [36]. By reducing oxidative and inflammatory signaling through A_2_A receptor stimulation, PDRN may protect gingival fibroblasts and promote mucosal regeneration in the context of bisphosphonate therapy. This lays the groundwork for potential future applications of PDRN in enhancing oral wound healing and maintaining soft-tissue integrity during antiresorptive treatment [37,38,39].

Despite these promising findings, this study comes with some limitations. Firstly, the experiments were conducted using the immortalized HGF-1 gingival fibroblast line only, which—although widely used as a model for oral soft-tissue biology—may not fully reflect the behavior of primary human cells. Therefore, future studies should confirm these findings in primary gingival fibroblasts and oral keratinocytes to establish cell-type specificity. Furthermore, in vivo models of oral mucosal injury or bisphosphonate-associated osteonecrosis are needed to better elucidate the physiological relevance and therapeutic potential of PDRN in preventing or alleviating MRONJ. Expanding these investigations could strengthen the translation of current in vitro findings into future clinical applications. In addition, our study compared the effects of PDRN with those of NAC, a well-established antioxidant. NAC primarily acts through direct scavenging of ROS and replenishment of intracellular glutathione stores, thereby suppressing oxidative stress [33]. In contrast, PDRN appears to exert its antioxidant effects indirectly via A_2_A receptor stimulation, which may lead to increased intracellular cAMP levels and the activation of downstream anti-inflammatory signaling cascades that subsequently suppress ROS generation and kinase activation. This distinction suggests a broader cytoprotective potential of PDRN, which may modulate not only oxidative stress but also inflammatory and survival signaling pathways [39].

## 4. Materials and Methods

### 4.1. Cell Culture

The human gingival fibroblast (HGF)-1 cell line was obtained from the American Type of Cell Culture (Catalog No. CRL-2014^TM^). HGF-1 cells were cultured in Dulbecco’s Modified Eagle’s Medium (DMEM) (Welgene, Daegu, Republic of Korea) supplemented with 10% heat-inactivated fetal bovine serum (Gibco, Grand Island, NY, USA) and 1% penicillin/streptomycin (Welgene) at 37 °C in a humidified atmosphere of 5% CO_2_.

### 4.2. Materials

Zoledronic acid (ZA) was purchased from Sigma-Aldrich (Cat. No. SML0223) (St. Louis, Mo, USA). Primary antibodies for phosphorylated p-PKB (S473) (cat. no. 9271), PKB (cat. no. 9272), p-TBK1 (S172) (cat. no. 5483), and TBK1 (cat. no. 3504) were obtained from Cell Signaling Technology, Inc. (Beverly, MA, USA). Primary antibodies for p-STAT-3 (Y705) (cat. no. sc-8059) and STAT-3 (cat. no. sc-8019) as well as small interfering RNA (siRNA) of control (cat. no. 37007), STAT-3 (cat. no. 29493), TBK1 (cat. no. 39058) or PKB (cat. no. 29195), were obtained from Santa Cruz Biotechnology (Dallas, TX, USA). An anti-actin primary antibody was obtained from Sigma-Aldrich (St. Louis, Mo, USA). The adenosine A_2_A receptor (A_2_AR) antagonist ZM241385 was purchased from MedChemExpress (Cat. No. HY-19532, Monmouth Junction, NJ, USA). Control siRNA (cat. no. sc-37007), TBK1 siRNA (cat. no. sc-39058), STAT-3 siRNA (cat. no. sc-29493), PKB siRNA (cat. no. sc-29195) were obtained from Santa Cruz Biotechnology (Dallas, TX, USA). N-acetyl-L-cysteine (NAC) (cat. no. A9165) was purchased from Sigma-Aldrich (St. Louis, MO, USA). A modified radio-immunoprecipitation assay buffer (Sigma-Aldrich; Merck) containing proteinase inhibitor cocktail (PIC) (1×) and nitrocellulose membranes were obtained from Millipore (Bedford, MA, USA). PDRN (CELLVANE Inj.) was obtained from Zerone Cellvane, Inc. (Cheonan, Chungcheongnam-do, Republic of Korea).

### 4.3. Cell Count Analysis

HGF-1 cells (2 × 10^4^ cells/500 µL/well) were seeded in a 24-well plate overnight. Cells were treated with ZA in the absence or presence of PDRN or ZM241385 at the indicated concentrations and times. Cells were then washed twice with PBS. The number of survived cells, that cannot be stained with trypan blue dye (0.4%, cat. no. 15250-061, Gibco, Grand Island, NY, USA), was counted using a phase-contrast microscope. The cell count assay was performed in triplicate. Data are mean ± standard error (SE) of three independent experiments. Survival is expressed as a percentage of control.

### 4.4. Preparation of Whole-Cell Lysates

After treatments, HGF-1 cells were washed twice with PBS containing 1 mM Na_3_VO_4_ and 1 mM NaF and subsequently exposed to cell lysis buffer [20 mM Tris-Cl (pH 7.5), 150 mM NaCl, 1 mM EDTA, 1 mM EGTA, 1% NP-40, 1% sodium deoxycholate, 2.5 mM sodium pyrophosphate, 1 mM β-glycerophosphate, 1 mM sodium vanadate, 1 mg/mL leupeptin, 1 mM phenylmethylsulfonyl fluoride. The cells were then harvested and centrifuged for 15 min at 4 °C and 13,000× *g*. The supernatant was extracted, and its protein concentration was determined by a bicinchoninic acid (BCA) protein assay kit (Thermo Scientific, Rockford, IL, USA) at 560 nm using a microplate reader (Bio-Rad Laboratories, Inc., Hercules, CA, USA).

### 4.5. Western Blot Analysis

Proteins (50 µg) were run and separated by sodium dodecyl sulphate-polyacrylamide gel electrophoresis and transferred onto nitrocellulose membranes (Millipore). The membranes were blocked with 5% (*w*/*v*) skim milk in Tris-buffered saline (TBS) containing 0.1% Tween 20 (TBST) for 2 hours (h). The membranes were incubated with specific antibodies at 4 °C overnight. The membranes were then washed twice with TBST and exposed to secondary antibodies conjugated to horseradish peroxidase (HRP) for 2 h at room temperature, and the immunoreactivity was detected by Western Bright^TM^ Enhanced chemiluminescence (ECL) reagent (cat. no. K-12045-D20) was purchased from Advansta Corporation (San Jose, CA, USA). Actin and each total protein corresponding to each phosphorylated protein counterpart, were used as an equal protein loading control.

### 4.6. siRNA Transfection

HGF-1 cells were seeded at a density of 1 × 10^5^ cells per well in 6-well plates or 5 × 10^4^ cells per well in 24-well plates, 24 h prior to transfection. Cells were transfected with either 100 pM of control siRNA (siCon) or target-specific siRNAs, including TBK1 siRNA (siTBK1), PKB siRNA (siPKB), or STAT-3 siRNA (siSTAT-3), using Lipofectamine^®^ RNAiMAX Transfection Reagent (Invitrogen, Waltham, MA, USA) according to the manufacturer’s protocol. Transfection was carried out for 6 h in Opti-MEM medium, after which the medium was replaced with complete DMEM supplemented with 10% FBS. Cells were then incubated for an additional 18 h, resulting in a total transfection period of 24 h. For TBK1 knockdown experiments, cells were subsequently treated with ZA (50 µM) for 48 h. In contrast, cells used for PKB and STAT-3 knockdown experiments were harvested immediately following the 24-h transfection period. The experimental groups included were non-transfected control (NT), siCon, siTBK1, siPKB, and siSTAT-3. Transfection efficiency and gene silencing were confirmed by Western blot analysis using antibodies against total TBK1, PKB, and STAT-3. In addition, morphological changes and cell viability were assessed via phase-contrast microscopy and a cell counting assay.

### 4.7. Measurement of Intracellular ROS

The generation of ROS was measured by an inverted fluorescence microscope (Olympus Life Science, Shinjuku, Tokyo, Japan) using 2′, 7′-dichlorofluorescein-diacetate (DCFH-DA) as a substrate. Briefly, HGF-1 cells were grown in 12 well cell culture plates (*N*= 3) at a density of 0.1 ×106 cells/1 mL/well overnight. Cells were treated with or without ZA at a concentration of 50 µM for 1, 2, 8, or 24 h. In addition, cells were co-treated without or with ZA, NAC (5 mM) and PDRN at 100 µg/mL for 8 h. Then after 8 h, cells were loaded with DCFH-DA to a final concentration of 20 µM for 20 min. HGF-1 cells were washed twice with PBS. The ROS generation was measured by DCF fluorescence at excitation 488 nm and emission 525 nm intensity was quantified using Image J software (version 1.53, NIH, USA). with results expressed as percentage intensity relative to controls. All experiments were performed in biological triplicate (*N* = 3) with three technical replicates per experiment.

### 4.8. Apoptosis Detection by FITC Annexin V with 7-AAD and Flow Cytometry

HGF-1 cells were seeded at a density of 1 × 10^5^ cells per well in 6-well plates and incubated overnight to allow for cell attachment. Cells were then treated for 48 h with one of the following conditions: untreated control, PDRN (100 µg/mL), ZA (50 µM), or a combination of ZA and PDRN. Following treatment, both floating and adherent cells were collected by trypsinization and centrifugation, washed twice with cold PBS, and resuspended in 1× Annexin V binding buffer. Apoptosis was assessed using the FITC Annexin V Apoptosis Detection Kit with 7-AAD (BioLegend, San Diego, CA, USA)**,** following the manufacturer’s instructions. Briefly, cells were incubated with FITC Annexin V and 7-AAD for 15 min at room temperature in the dark, diluted with binding buffer, and analyzed within 1 h using a BD FACSCalibur^TM^ flow cytometer (BD Biosciences, San Jose, CA, USA). A minimum of 10,000 events was collected per sample, and data were analyzed with FlowJo™ software (version 10, BD Biosciences). Cells were classified as viable (Annexin V^−^/7-AAD^−^), early apoptotic (Annexin V^+^/7-AAD^−^), late apoptotic (Annexin V^+^/7-AAD^+^), or necrotic (Annexin V^−^/7-AAD^+^). All experiments were conducted in biological triplicate.

### 4.9. Statistical Analysis

Cell count analysis and western blot experiments were measured in triplicate and repeated three times. The results were expressed as mean ± standard error (SE). One-way ANOVA followed by Sidak’s post hoc test was used to compare the significance of the difference. All significance testing was established on a *p*-value of <0.05.

## 5. Conclusions

Our study demonstrates that ZA inhibits the growth of HGF-1 cells by downregulating the pro-survival kinase PKB and upregulating the stress-responsive kinase TBK1. PDRN effectively counteracts ZA-induced cytotoxicity in HGF-1 cells by attenuating the hyperactivation of TBK1. These findings provide a mechanistic rationale for the potential clinical application of PDRN in the prevention or management of ZA-induced oral mucosal complications, particularly those involving the loss of gingival fibroblasts. Furthermore, this work underscores the need for continued investigation into the therapeutic utility of PDRN in this context.

## Data Availability

Data will be made available on request.

## Supplementary Materials

The following supporting information can be downloaded at: https://www.mdpi.com/article/10.3390/ijms262311367/s1.
