# Supplementary material for: The Mitigating Effect and Mechanism of Polydeoxyribonucleotide Against Zoledronic Acid-Induced Growth Suppression of Human Gingival Fibroblasts
Source: Int J Mol Sci. 2025 Nov 24;26(23):11367. doi: 10.3390/ijms262311367 (PMC12692050; doi:10.3390/ijms262311367)
Supplement: Supplementary file 1 [file ijms-26-11367-s001.zip › ijms-3933503-supplementary.pptx]

## Slide 1
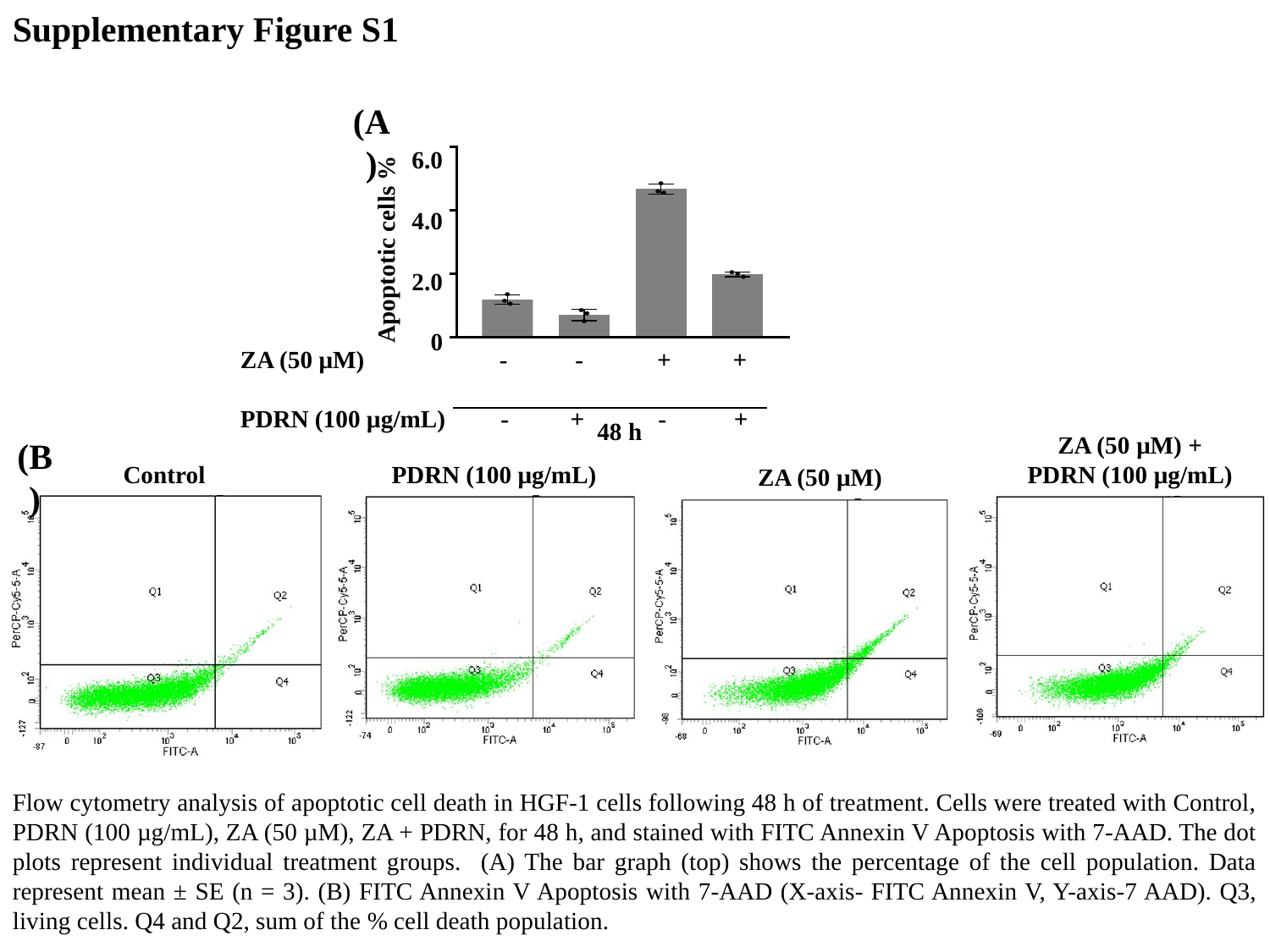

Supplementary Figure S1
(A)
6.0
4.0
2.0
0
Apoptotic cells %
ZA (50 μM) - - + +
PDRN (100 µg/mL) - + - +
48 h
ZA (50 μM) +
PDRN (100 μg/mL)
(B)
Control
PDRN (100 μg/mL)
ZA (50 μM)
Flow cytometry analysis of apoptotic cell death in HGF-1 cells following 48 h of treatment. Cells were treated with Control, PDRN (100 µg/mL), ZA (50 µM), ZA + PDRN, for 48 h, and stained with FITC Annexin V Apoptosis with 7-AAD. The dot plots represent individual treatment groups. (A) The bar graph (top) shows the percentage of the cell population. Data represent mean ± SE (n = 3). (B) FITC Annexin V Apoptosis with 7-AAD (X-axis- FITC Annexin V, Y-axis-7 AAD). Q3, living cells. Q4 and Q2, sum of the % cell death population.
